# Supplementary material for: An open-source framework for large-scale, flexible evaluation of biomedical text mining systems
Source: J Biomed Discov Collab. 2008 Jan 29;3:1. doi: 10.1186/1747-5333-3-1 (PMC2276192; doi:10.1186/1747-5333-3-1)
Supplement: Additional file 1 — Evaluation platform source code. The additional file contains the software evaluation framework discussed in this paper. The most current release can be downloaded from SourceForge.net [1]. See the accompanying README file for instructions on installation and use. Also included in the distribution is a collection of UIMA wrappers for some commonly used BioNLP tools and annotated corpora. [file 1747-5333-3-1-S1.zip › bionlp_uima_repository-1.0.1/docs/api/src-html/edu/uchsc/ccp/util/nlp/stemmer/PorterStemmer.html]

```
001    package edu.uchsc.ccp.util.nlp.stemmer;
002    
003    /**
004     * Extends the original Porter Stemmer implementation by Martin Porter (See {@link org.tartarus.PorterStemmer}) to accept String input.
005     * 
006     * @author William A Baumgartner, Jr.
007     * 
008     */
009    public class PorterStemmer extends org.tartarus.PorterStemmer {
010    
011        /**
012         * Stems the input text
013         * 
014         * @param inputText
015         *            text to stem
016         * @return the stemmed text using the Porter algorithm
017         */
018        public String stem(String inputText) {
019            inputText = inputText.toLowerCase();
020            add(inputText.toCharArray(), inputText.toCharArray().length);
021            stem();
022            return toString();
023        }
024    
025    }
```
